# Laser Assisted Non-surgical Periodontal Therapy: A Double Blind, Randomized Clinical Trial

**DOI:** 10.2174/1874210601711010079

**Published:** 2017-02-14

**Authors:** Joseph D. Everett, Jeffrey A. Rossmann, David G. Kerns, Ibtisam Al-Hashimi

**Affiliations:** Department of Periodontics, Texas A&M University Baylor College of Dentistry, Dallas, TX 75246, USA

**Keywords:** Carbon dioxide laser, Microbial biofilm, PCR analysis, Scaling and root planing, Laser therapy

## Abstract

The objective of this study was to examine potential benefits of using laser therapy for secular decontamination in conjunction with scaling and root planing in the treatment of chronic periodontitis. The study was performed on 173 teeth in 14 patients in a split-mouth design, one side received scaling and root planing followed by laser therapy using a carbon dioxide (CO_2_) laser with an ablative handpiece (test group); the contralateral side received scaling and root planing without laser (control group). Clinical and laboratory parameters were evaluated prior to treatment and at 3 and 6 months following therapy; clinical measurements were performed by two blinded examiners. The clinical parameters included measurement of gingival recession (REC), bleeding on probing (BOP), clinical attachment level (CAL), pocket depth (PD), furcation involvement (FUR), and tooth mobility (MOB). Laboratory testing to determine the levels of periodontal pathogens was performed using PCR techniques. The results of the study revealed statistically significant differences in clinical and laboratory parameters at 3 and 6 months after therapy for both test and control groups, but no significant difference was observed between the two groups. However, sites receiving laser therapy tended to show a greater decrease in probing depths, gain in clinical attachment level, and reduced bacterial levels. In conclusion, the overall results of the study suggest a potential benefit of using laser therapy in conjunction with scaling and root planing for the treatment of chronic periodontitis.

## INTRODUCTION

The foundation of any periodontal therapy begins with the removal of bacterial plaque (biofilm) and calculus to allow healing of the periodontium. The difficulty of periodontal disease is that the periodontal microflora is extremely diverse. From Socransky’s work utilizing whole genomic DNA probes and checkerboard DNA-DNA hybridization to assess 13,261 plaque samples in 185 patients, a series of complexes were found to correlate well with the type of bacteria that colonize the biofilm [1]. The early colonizers are either independent of the defined complexes or members of the yellow (Streptococcus species) or purple complexes (Actinomyces species). These aerobic early colonizers lower the reduction-oxidation potential of the environment, facilitating the growth of anaerobic species.

Green, orange or red complexes have a propensity to be secondary colonizers. The green complex includes E. corrodens, Actinobacillus actinomycetemcomitans serotype a, and Capnocytophaga species. The orange complex includes Fusobacterium, Prevotella, and Campylobacter species. The red complex (*P. gingivalis*, *B. forsythus*, and *T. denticola*) is associated with bleeding on probing, an important clinical parameter of destructive periodontal diseases [1]. Another approach to determining the potential virulence of subgingival plaque in biofilm was shown in a preliminary study by Germano *et al*. using atomic force microscopy and analyzing the bacterial components at a nanoscale. Their work identified species of spirochetes, flagellated forms and filaments as representative of periodontal pathogens similar to those found in the green, orange and red complexes [2].

Recently laser therapy has been suggested as a potential tool to improve the outcome of non-surgical treatment of chronic periodontitis. However, the use of carbon dioxide lasers as an adjunct to non-surgical therapy has had conflicting results. Some suggest that CO_2_ laser could potentially damage root surfaces by resulting in deformed root surfaces [3]. Others suggest that the ablative effect of the CO_2_ laser on periodontally involved root surfaces may be beneficial [4, 5]. Another potential advantage of laser therapy is the possibility of decontaminating the periodontal sulcus [6]. Several *in vitro* studies have demonstrated the CO_2_ laser’s capacity to destroy bacteria at low energy density levels without detectable damage to the underlying root surface [7, 8]. Kojima *et al*. reported that the *in vitro* use of a CO_2_ laser killed more than 99% of *Porphyromonas gingivalis* (PG) and *Aggregatibacter actinomycetemcomitans* (AA) at 7.5 and 12.5 J/cm^2^ and significantly reduced LPS biological activity when irradiated by energy densities greater than 7.5 J/cm^2^ [9]. However, there is insufficient evidence to conclude if a laser can decontaminate a periodontal pocket [10]. The purpose of this study was to compare the outcome of scaling and root planing with and without CO_2_ laser for the treatment of chronic periodontitis, in a split mouth design; and to examine the effect of laser therapy on subgingival microbiota over a period of six months.

## MATERIALS AND METHODS

### Patients Population

The study protocol was approved by the Institutional Review Board at Texas A&M University-Baylor College of Dentistry. All subjects signed a written informed consent document prior to treatment. Inclusion criteria: Subjects were required to have a minimum of two contra-laterally similar periodontal probing depths (PD) ≥ 5mm with clinical attachment loss (CAL) ≥ 4mm for two or more teeth. Exclusion criteria: were patients with severe periodontitis as defined by McGuire’s criteria [11]; systemic diseases such as uncontrolled diabetes, uncontrolled hypertension, auto-immune diseases, *etc*. Patients requiring antibiotics prior to dental procedures or used medication such as antibiotics, steroids, anticoagulants, or anti-inflammatory agents within three months prior to treatment, pregnant/ lactating females, smokers (more than 10 cigarettes per day), individuals who had scaling and root planing within the past six months were also excluded. Out of 47 individuals screened, 14 patients met the inclusion criteria.

### Laser Therapy

**Fig. (1):**
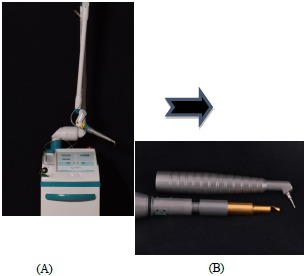
(a) Azuryt CTL 1401, CO_2_ (North American Clinical Laser LTD, Denver, CO). (b) Handpiece (Photonic Resources LTD, Denver, CO).

A CO_2_ laser (10.6 micron wavelength) with an ablative prototype handpiece (Photonic Resources, Denver, CO) was used, that allows for the laser beam to be focused directly into the sulcus (Fig. **1**). The treatment protocol involved the laser procedure to be performed in conjunction with scaling and root planing every 10 days for three appointments, following the initial therapy. This is in consensus with the protocol used by Kelbauskiene *et al*. where “the same procedure was performed once a week for each millimeter of pocket reduction desired to obtain a normal probing depth of 3 mm or less, which typically required an average of three appointments” [12]. This randomized, controlled clinical trial consisted of fourteen patients (5 male and 9 female) ages 34-65 (mean 54 years) recruited from the patient pool at Texas A&M University Baylor College of Dentistry.

#### Bacterial Sampling

Bacterial samples were taken from the 4 deepest probing depths (2 test sites and 2 control sites); the same sites were used throughout the study. The sites were isolated with cotton rolls, dried with air, and had supragingival plaque removed by a curette. Sterile endodontic paper points were inserted to depth and remained for 30 seconds. Samples were immediately placed into a sterile micro-centrifuge tube with 0.5 mL RNALater. Samples were frozen at -20°C until further analysis *via* multiplex PCR for presence of known periodontal pathogens by a commercial lab (OralDNA Labs (7400 Flying Cloud Drive, Suite 150, Eden Prairie, Minnesota 55344).

### DNA Extraction

Bacterial samples were pooled from 2 paper points, each was suspended into 0.5 mL of RNAlater (Qiagen, Germany). DNA extraction was performed through a combination of mechanical disruption of the bacterial cell and ion-exchange column purification. The 2 paper point and RNAlater solutions were combined to 1 vial and centrifuged at 10,620 RCF for 5 minutes. Approximately 900 μL of RNAlater supernatant were aspirated off and replaced with the same volume of 0.9% saline oral rinse solution, which was combined with 300 µL of zirconium beads and homogenized at 2500 rpm for 10 minutes (Tallboys High Throughput Homongenizer, Thermo-Fisher, Richardson, TX, USA). The resulting mixture was centrifuged, fractionated into 200 µL aliquot and purified using silica membrane (Qiacube HT DNA extractor; Qiagen, Germany).

### Analysis of Periodontal Bacteria

Automated PCR was performed using a CAS-4200 Robotic Workstation (Qiagen, Germany). Eleven bacterial species (Table **1**) were detected using asymmetric multiplexed polymerase chain reaction (PCR) with primers and molecular beacons designed to specific gene regions of each bacterial species. Three PCR reactions each contain primers and beacons specific for three bacterial species and the fourth reaction contains primers with molecular beacons for two species plus a set designed to amplify the human DNA sequence ApoB. Amplification and detection were performed using a Qiagen RotorGene (Qiagen, Germany). Parameters for read cycle and probe melt temperature were optimized for each bacterial species. Fluorescent emission resulting from molecular beacon hybridization was read at the determined read cycle of the PCR reaction and compared to the standard curve of known plasmid standards fluorescence to provide a semi-quantitative analysis of patient sample concentration for each bacterium. The calculated bacteria concentration of each species was compared to a clinical threshold concentration and reported as HIGH, LOW or NOT DETECTED (ND) relative to the clinical threshold. The ND range is determined by the limit of detection of each batch based on the fluorescence of the blank controls (noise). In general, the ND range is ~10^3 copies/mL and below. The low range is any signal between the ND range (~10^3 c/mL) and the high value. For a bacterial load to be considered high, the concentration must be greater than the following values: AA ≥ 10^4^ c/mL, the red complex (PG, TG, TD) ≥ 10^5^ c/mL and remaining bacteria ≥ 10^6^ c/mL.

### Clinical Parameters

At the initial visit, blinded examiners (JR or DK) made clinical measurements at each time point (0, 3 and 6 months). Measurement of the depths of the periodontal sulcus (PD) measured with a UNC 15 periodontal probe to the nearest 1 mm increment. A single UNC 15 probe was used for all examinations. Six measurements were made around each tooth involved in the study: mesio-facial and lingual, mid-facial and lingual, and disto-facial and lingual surfaces. Recession (REC), bleeding on probing (BOP), furcation involvement (FUR), and mobility (MOB) were also recorded.

At the completion of the initial measurements, scaling and root planing (S/RP) was performed under local anesthesia on all sites greater than 4 mm. All S/RP were completed in one session without time constraint by one examiner with ultrasonic scalers and hand instruments (JE). Immediately following S/RP, one side (right *vs*. left) was randomly assigned to the test or control group (split-mouth design) using a coin flip. The control side did not receive any additional treatment except for a sham pass with the laser handpiece so as to prevent possible patient bias. To decontaminate the gingival margin of the test side, the tip of the handpiece is dragged along the gingival sulcus, taking care to keep the tip parallel to the long axis of the tooth. This initial pass is at a power setting of 4 watts in continuous mode. The prototype handpiece conditions the beam to specifically ablate and deliver a power density of approximately 280 W/cm^2^ through a “tip” with an internal diameter of 0.762 mm in continuous mode. This equates to approximately 25 - 50 J/cm^2^ at 4 watts.

The second pass is intended to decontaminate and ablate the periodontal pockets. The setting is increased to 8 watts continuous mode to deliver a power density of approximately 561 W/cm^2^ (75 - 100J/cm^2^ at 8 watts) and the tip of the handpiece is placed into the pocket. Care is again taken to avoid using the CO_2_ laser on hard tissue or mucosa by maintaining a parallel orientation to the long axis of the tooth to the base of the probing depth. Starting in the distal portion of the pocket, the laser was continuously “dragged” in the pocket and took approximately 2 seconds to “drag” the tip from distal to the mesial portion. The laser tip is also “tipped” at approximately 45° interproximally in an attempt to decontaminate the interproximal portion of the pocket. The motion of the laser tip is similar to the walking of a periodontal probe in the pocket during a periodontal exam; however, the tip is dragged along the depth of the pocket during the entire second pass. This drag is done once on the buccal and once on the lingual portion of the pockets for a total irradiation time of 4 seconds per tooth. After the second pass, damp gauze is used to place firm pressure on the gingiva to allow a clot to develop. Patients were asked to use analgesics for pain. Oral hygiene instructions were reviewed and demonstrated which included proper use of floss and the modified Stillman brushing technique.

The patients returned at 10, 20, and 30 days post-scaling for supragingival prophylaxis, oral hygiene instructions, and additional laser therapy to the test side in an effort to block epithelial down growth on the root surface and decontaminate the pocket, using a previously published protocol [12]. Patients were evaluated at 3 months and 6 months post-scaling. Bacterial sampling and clinical measurements were repeated at 3 and 6 month appointments, and the patients received a supragingival prophylaxis. Patients that presented with probing depths ≥ 5 mm at the final evaluation were referred for further treatment options.

### Statistical Analysis

Each site had clinical measurements at baseline, three month, and six month time points: which included probing depth (PD), gingival recession (VR) [converted to clinical attachment level (CAL)], bleeding on probing (BOP), Miller mobility Scores, Furcations (Glickman), and modified O’Leary Plaque Index (PI), which is given as percent of plaque free sites. For variables measured at the three time points, a longitudinal approach for nonparametric and parametric data was used to analyze the data according to group classification and for PD greater than 5 mm, assuming an unstructured covariance matrix, and a mixed effect between time and the variable of interest. BOP was measured as percent of sites bleeding for longitudinal data analysis. Time was also treated as an ordinal variable as opposed to a continuous linear since not all patients are measured at the same time. Additionally, the model with ordinal time proved to be a better fit [observing the AIC and the -2 log (likelihood)].

Results were tabulated and analyzed as described above using SAS 9.3 and R, in particular prewritten functions such as proc mixed with proc ranked to use Friedman’s method, proc glimmix (for BOP), and proc univariate for all variables to test for normality.

## RESULTS

### Probing Depths

The study population consisted of 9 females and 5 males, ages range 34-65 years (median 54 years). At baseline the mean pocket depth was 4.04 ± 0.060 mm and decreases to 3.25 ± 0.051 mm for both groups at the 3 month mark (p = 0.035). At 6 months, there was further decrease to 3.05 ± 0.044 mm (p = 0.022) for both groups. At baseline, the mean pocket depth was 4.16 ± 0.086 mm for experimental sites and 3.93 ± 0.083 mm for controls. From baseline to 3 months, a decrease of 0.80 ± 0.053 mm was observed for both groups with the test sites decreasing by 0.88 ± 0.076 mm compared to the control sites decrease of 0.71 ± 0.730 mm both were statistically significant (p < 0.05).

When an analysis was made for all *PD ≥ 5* mm, the overall baseline measurement starts at 5.7 ± 0.003 mm (T: 5.74 ± 0.073 mm *vs*. C: 5.65 ± 0.07 mm) and decreases to 4.04 ± 0.003 mm for both groups at 3 months (T: 4.01 ± 0.093 mm *vs*. C: 4.07 ± 0.105 mm) (p = 0.025). Probing depths further decreased to 3.77 ± 0.003 mm at 6 months (T: 3.72 ± 0.079 mm *vs*. C: 3.83 ± 0.085 mm) (p = 0.018). The control group’s probing depths, overall, are slightly deeper than the treatment group at the 3 and 6 months for *PD ≥ 5*. There was an overall decrease of 0.99 ± 0.051 mm in PD (p < 0.05). In comparing test to control sites, the test decreased by 1.14 ± 0.073 mm compared to the control’s 0.85 ± 0.070. When analyzing sites initially > 5 mm, a 2.02 ± 0.099 mm decrease was noted for the test compared to 1.42 ± 0.101 mm for control sites. There is no statistically significant difference between the treatment groups (intergroup) for *All Data* (p = 0.412) and *PD ≥ 5* over time (p = 0.131).

### Clinical Attachment Level

Table **2**, overall (*All Data)* indicates no significant difference in response between the two groups, except at baseline. At baseline the CAL is 4.03 ± 0.084 mm for the test group and 3.72 ± 0.079 mm for the control group. Both groups improve to 3.24 ± 0.053 mm at three months. The levels further improve between three months and six months by 0.24 ± 0.050 mm to 3.05 ± 0.046 mm. When focusing on sites with *PD ≥ 5,* the data shows that there is a sharp decrease in CAL between 5.21 ± 0.003mm baseline to 3.83 ± 0.004 mm (p = 0.018) at the three month visit [3.90 ± 0.105 mm for control (p = 0.021) and 3.77 ± 0.103 mm for test (p = 0.015)]. Between the three month and six month visit there is another decrease of 0.28 ± 0.004 mm to 3.56 ± 0.003 mm (p = 0.010).

When focusing on sites with PDs ≥ 5 mm initially, the CAL test sites improved from baseline to 6 months by 1.83 ± 0.107 mm compared to the control group’s improvement of 1.44 ± 0.103 mm both are statistically significant changes (p < 0.05). However, there is no statistically significant difference between the treatment groups for both *All Data* and *PD ≥ 5* (p = 0.44 and p = 0.78 respectively).

As for BOP, PI, Mobility and Furcation involvement there was no difference at baseline between groups. Although the sites improved significantly from baseline in regards to the clinical parameters, there was no statistically significant difference between treatment modalities (Table **2**). The changes in REC went from -0.17 mm overall to -0.13 mm overall at 6 months showing no differences among groups. BOP was initially 69% of all sites overall and reduced by 29% at 6 months with little difference between test and control sites. Both sides showing general improvement from therapy, is 40% overall at 6 months. PI was 21% plaque free surfaces at baseline overall and improved to 62% plaque free surfaces overall at 6 months, with little differences in this split mouth study. Neither parameter of BOP nor PI reached the levels acceptable for good plaque control according to therapeutic standards.

### Bacterial Analysis

The bacteria are segregated into the complexes as described by Socransky [1]. Figs. (**2** - **3**) black lines are present to delineate boundaries set for a bacterial load to be considered N/D, Low, or High. Columns ending below the 3.00 line are considered N/D. Any column ending between the 3.00 line and the superior line (at 4.00, 5.00, and 6.00) is in the Low detection range. Columns ending above the line are considered to have a High bacterial load. The overall median baseline values are plotted in Fig. (**2**) which shows that both groups present with high detectable levels of PG, TF, and CR. The control group had a higher percentage of EN compared to the higher levels of AA and FN noted in the test group. The medians of each calculated bacterial level are plotted against the time points of interest in Figs. (**3** and **4**) in which generalized trends can be visualized.

In an attempt to simplify the data as much as possible, the bacteria will be addressed *via* complexes overall per treatment group. When analyzing between test and controls, Table **5** depicts changes from Baseline to 3 months and Baseline to 6 months for both groups. The trends noted suggest that there is an overall decrease in the amount of Red and Orange complex bacteria, but an initial increase in the Green Complex which subsequently decreases from the 3 to 6 month mark leading to a final overall decrease. The control group tended to have a better response to AA, TF, CR, and CS while the test group seemed to have a greater reduction in the amount of PG, TD, EN, FN, PI, PM and EC.

### The Red Complex: *Porphyromonas gingivalis* (PG), *Tannerella forsythia* (TF), *Treponema denticola* (TD)

**Fig. (2):**
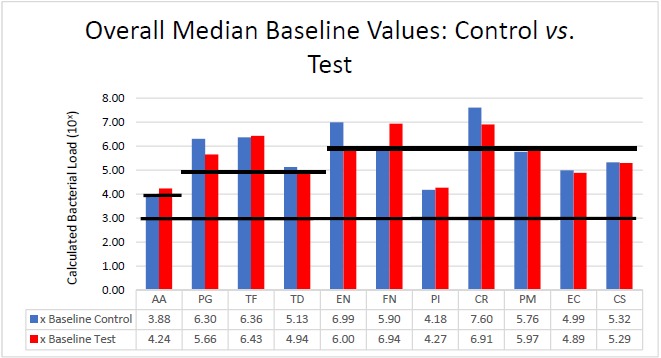
Bacteria identified in test and control sites at baseline. Horizontal black lines represent not detectable (ND) levels (below 10^3^), low levels (10^3^ - 10^6^), and high levels ≥ 10^6^ compared to standard.

**Fig. (3):**
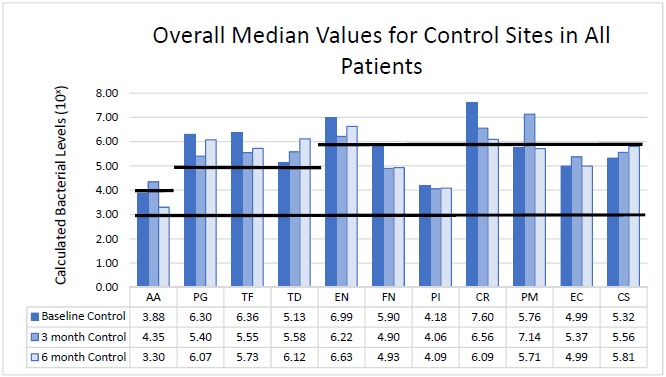
Comparison of median bacterial levels at baseline *vs*. 3 months and 6 months following treatment for control group.

**Fig. (4):**
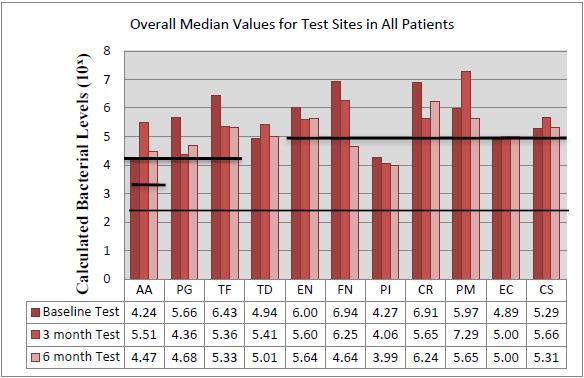
Comparison of median bacterial levels at baseline *vs*. 3 months and 6 months following treatment for test group.

Addressing the control and test sites overall, both groups have high levels of PG, TF, and TD at baseline (Fig. **2**). An appreciable decrease of PG and TF can be seen from baseline levels to the 3 month sample in both groups (Figs. **3** and **4**). However, in Fig. (**3**), although the control group had a decrease in PG and TF at 3 months, the levels are still considered in the high bacterial load category. Interestingly, in the control group, TD actually increased throughout the study. In the test group from Fig. (**4**), both PG and TF decreased, with PG entering the low detection limit in the 3 and 6 month mark. TF decreased from baseline and maintained a comparable drop at 6 months, but the overall level is still considered high. Another interesting point is TD increasing from a low detection level at baseline (4.94) to a high detection limit at 3 months, only to drop again slightly to a near low detection level (5.01) at 6 months.

### The Orange Complex: *Eubacterium nodatum* (EN), *Fusobacterium nucleatum/periodonticum* (FN), *Prevotella intermedia* (PI), *Campylobacter rectus* (CR), *Peptostreptococcus micros* (PM)

From Fig. (**3**), EN and CR are the only bacteria noted at a high limit at baseline for the control group. EN decreased slightly at 3 months, but rebounded at 6 months all while still considered in a high bacterial load category. CR dropped drastically at 3 months and continued at 6 months, but remains in the high load group overall. FN initially started in the low category, but decreased at 3 months and remained nearly consistent at 6 months. PI remained nearly consistent throughout the study in the low detection category. PM interestingly increased dramatically from baseline to 3 months, but returned to near baseline values at 6 months.

Fig. (**4**), EN dropped from baseline to 3 months and remained consistent at 6 months for the test sites. FN initially started in a high category (6.94) drops at 3 months and continued to decrease even further, entering the low detection category at 6 months. PI remained nearly consistent throughout the study. CR decreased initially, but rebounded to high detection levels at 6 months. PM, consistent with the trend noted in the control sites, increased drastically from baseline to 3 months, but dropped again to below baseline levels at 6 months.

### The Green Complex: *Aggregatibacter actinomycetemcomitans* (AA), *Eikenella corrodens* (EC), *Capnocytophaga* species (*gingivalis, ochracea, sputigena*) (CS)

As seen in Fig. (**3**), the control group had AA increase from low to a high level at 3 months, but dropped below baseline levels at 6 months. EC trended similarly while CS consistently increased throughout the study, but both EC and CS remained within the confines of the low detection limit. From Fig. (**4**), the test group responded like the control group, but at higher spike in AA is seen from baseline to 3 months that remained higher than baseline levels at 6 months.

### Overall Analysis Between Groups

When analyzing between test and controls, Fig. (**5**) depicts changes from Baseline to 3 month and Baseline to 6 month for both groups. The trends noted suggest that there is an overall decrease in the amount of Red and Orange complex bacteria, but an initial increase in the Green Complex which subsequently decreases from the 3 to 6 month mark leading to a final overall decrease. The control sites tended have a better response to AA, TF, CR, and CS while test sites seemed to have a greater reduction in the amount of PG, TD, EN, FN, PI, PM and EC. For this study, LANST performed better in reducing PG, EN, FN, PM, EC while S/RP alone had better results in reducing TF and CS; however, no statistical significance was found.

## DISCUSSION

In this randomized, controlled clinical trial a novel approach was utilized in an attempt to investigate the adjunctive use of a CO_2_ laser subgingivally in the non-surgical treatment of moderate to severe, chronic periodontitis. The rationale of the study was to test the theory that laser application to the periodontal pocket during treatment with scaling and root planing would improve the clinical and microbial outcome in a favorable manner over a six-month evaluation period. Within the confines of this study, the adjunctive use of CO_2_ laser decontamination was not clinically nor statistically significantly better than scaling and root planing alone for the treatment of moderate to severe, chronic periodontitis in selected teeth when assessing clinical parameters. However, LANST had a propensity to reduce PG, EN, FN, PM, EC bacterial levels more than S/RP alone over a six month period when analyzed by multiplex PCR analysis. This includes many of the periodontal pathogens implicated in active disease states.

The non-surgical results from this study are in agreement with other non-surgical studies. After non-surgical therapy, Morrison *et al*. reported a 0.96mm pocket depth reduction in sites with initial probing depths of 4-6mm [13]. Kaldahl *et al*. reported sites with initial probing depths from 5.0-6.0mm had a 1.23mm reduction in probing depths with 0.96mm gain in clinical attachment 3 months afterwards [14]. Pope *et al*. saw a probing depth reduction of 1.8 mm (1 mm gain in clinical attachment) overall in their study using a CO_2_ laser for de-epithelialization only in combination with S/RP, but with an increase in recession [15]. In this study, when combining the test and control sites, there was a 1.51 ± 0.003 mm overall reduction in probing depth (1.28 ± 0.004 mm clinical attachment gain) for sites with initial probing depths ≥ 5 mm from baseline to 6 months (Table **2**). One interesting thing to note is that in sites with probing depths ≥ 5 mm at baseline, the laser group had a slightly better gain of CAL of 1.46 ± 0.105 mm compared to the control sites at 1.09 ± 0.104 mm. This average difference of nearly 0.4 mm may be considered a moderate benefit for adjunctive therapy when utilizing the criteria from the recent systematic review of non-surgical periodontal therapy [16, 17]. However, this needs to be verified with larger sample sizes with better plaque control between visits. Several recent reports of Nd: YAG, Er: YAG, and Er, Cr: YSGG lasers involve inserting a laser tip into the sulcus so that the laser irradiates the sulcular epithelium and root surface. Mullins *et al*. used a third generation CO_2_ laser with a handpiece and tip allowing subgingival application into the periodontal pocket. She energized the pocket in one session only using an exposure of 37.5 J/cm^2^ and found 71% of the bacterial count analyses for the eight periodontal pathogens evaluated remained the same. Her conclusion was that a one-time use of the CO_2_ laser inside the periodontal pockets did not sterilize nor substantially reduce the bacterial population [18]. However, the results of this current study are in agreement with studies that report positive gains from laser therapy inside the sulcus, but not statistically significantly superior to S/RP alone [19].

Several factors may account for this. Although Breininger *et al*. states that a single session of S/RP can yield a significant reduction in bacterial populations even without complete removal of all sub-gingival calculus; it is possible that plaque control was a factor in the outcome of this study [20]. Plaque index was recorded and by having the patients return every 10 days for plaque control and prophylaxis for the first month after initial therapy, a general trend was noted for a better result for the side receiving the LANST protocol during this first month. However, an observation from the 3 to 6 month time frame was an increase in detrimental clinical parameters as seen with an increase in BOP in patients with a lack of ideal plaque control. With the noted increase in plaque scores seen for all the patients, this lack of oral hygiene can be a critical deterrent of healing with neither group reaching the ideal 85% plaque free percentage at any time point. This can be supported by the literature showing “both surgical and non-surgical methods of treatment are effective in eliminating gingivitis and reducing probing depths provided the subgingival plaque is eliminated and re-infection prevented following active therapy” [21].

Another factor is the relatively small sample size (n = 14). However, the report of PCR analysis for known periodontal pathogens is advantageous in seeing any possible changes to the periodontal environment throughout the study. However, several other drawbacks must be considered. Although the PCR analysis will detect bacterial RNA within the sulcus, there is no way to differentiate between live, thriving bacteria or just bacterial remnants present within the sulcus. Considering the split mouth design, there is the possibility of cross over contamination from sites that received only S/RP which could “re-infect” LANST sites. The recurrence of several periodontal pathogens after 3 months appears to be in consensus with the literature. Magnusson reported that in the absence of oral hygiene, spirochetes and motile rods were reestablished in 4 to 8 weeks [22]. Mousques observed that after a single session of S/RP, without proper oral hygiene, there was a return to baseline values by 3 months [23]. In a study of 12 patients with moderate probing depths (4-6 mm), Tabita *et al*. noted the development of subgingival plaque within 14 days, even with daily professional care [24]. A future design could be a case controlled study that allows for matched subjects to undergo either S/RP or LANST as a full mouth therapy. It must also be noted although several bacteria in the study appeared to decrease over the time; some (TF and AA) were very resilient and maintained high values at multiple time points for both groups. PM even increased from baseline to 3 months, just to return to near baseline levels at 6 months. There was a trend for nearly all bacterial species except AA, TD, PM, EC and CS to decrease after LANST at 3 months. In this study, the LANST protocol was only performed during the first month. It would be interesting to see if these downward trends would continue if LANST was performed at each 3 month periodontal maintenance appointment.

Another observation noted during the study was that patients tended to report less sensitivity on the side that had received the LANST protocol, but no attempt was made to officially survey the patients’ subjective responses to therapy. In addressing the subjective decrease in sensitivity by the patient, future research would include a visual analog scale, and evaluate possible surface changes to the root surface, as found in the Mullins *et al*. study [18]. From the literature, Pogrel *et al*. found that when using a Xanar Articulator CO_2_ laser with a 1 mm focused lens at 17.5 W (2320 W/cm^2^), the tissue necrosis lateral to the incision line was dependent on the water content of the tissue. They report a mean width of necrosis lateral to the incision was 85.9 μm for epithelium, 51.1 μm for loose connective tissue, and 96.1 μm for dense connective tissue [25]. Although the LANST protocol uses a conditioned ablative beam, it is uncertain if the beam may cause any changes on the root surface. In a study by Almehdi *et al*., the authors found that direct irradiation of a root surface at 1.0 W without coolant in a non-contact focused mode for 2 seconds, the histological and scanning electron micrographs of the surface revealed “melted and resolidified structures with numerous major and minor microcracks” [26].

One possible explanation for reduced sensitivity after scaling and root planing plus laser ablation may be answered by Barone *et al*. The authors subjected extracted root surfaces to different modes of CO_2_ laser beams in an *in vitro*, scanning electron microscope study. When comparing an 8 W, continuous mode with a focused beam of 0.8 mm to a 2 W, pulsed mode at 4 Hertz, non-focused beam of 4 mm aligned directly to the root, the defocused mode did not result in the same amount of damage to the root surface. While the continuous mode created craters and fissures, the defocused beam created smooth, flat surface that sealed the dentinal tubules [3]. Ideally the tip is kept parallel to the root surface, but the heat may be decontaminating and sealing dentinal tubules, resulting in desensitization of the root surface.

In regards to the potential for root surface changes from the application of the carbon dioxide laser, although the ablative hand piece was designed to minimize direct heat distribution to the adjacent root surface, only a direct observation of extracted teeth under ultrastructural examination can verify the effects. In this study, no teeth were extracted for examination of possible root damage.

Further research is needed to evaluate the efficacy of CO_2_ laser therapy as an adjunct to non-surgical therapy. For future studies, the authors recommend use in patients with established plaque control during maintenance appointments in residual probing depths ≥ 5 mm or sites with consistent BOP. It is also recommended to include the use of a visual analog scale to account for subjective responses in regards to sensitivity or discomfort during or after therapy. Additionally, full mouth debridement with the laser appears to be favorable to a split mouth design to better reduce the effect of cross-contamination.

## CONCLUSION

Within the limitations of this six month study, sites treated with the LANST procedure tended to show a greater decrease in probing depths and greater gains in clinical attachment levels. However, the results were not statistically significantly better than scaling and root planing alone. The decrease in several suspected periodontal pathogens for the first 3 and 6 months after therapy suggests a potential benefit for using laser therapy as adjunct tool for non-surgical treatment of chronic periodontitis. However, further research is needed to evaluate the efficacy of the LANST protocol in larger clinical trials.

## Figures and Tables

**Fig. (5):**
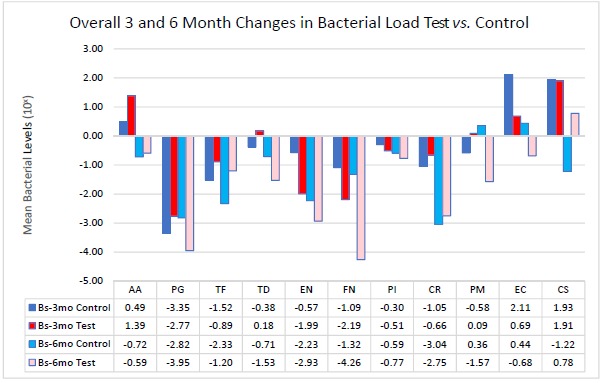
Overall changes in bacterial load among test and control groups over 3 and 6 months following periodontal therapy.

**Table 1:** Bacterial species detected using asymmetric multiples polymerase chain reaction (PCR).

Reaction A	Reaction B	Reaction C	Reaction D
*Actinobacillus actinomycetemcomitans*	*Prevotella Intermedia*	*Campylobacter rectus*	*Fusobacterium nucleatum/periodonticum*
*Eubacterium nodatum*	*Capnocytophaga spp. (gingivalis, ochracea, sputigena)*	*Tannerella forsythia*	*Treponema denticola*
*Porphyromonas gingivalis*	*Peptostreptococcus micros*	*Eikenella corrodens*	Internal Control: Apolipoprotein B

**Table 2:** Clinical parameters for test and control group at baseline, 3 months and 6 months. Mean values are represented for all data and sites with PD≥5mm.

			All Data				PD≥5mm	
	PD (mm)	CAL (mm)	Recession (mm)	BOP (% bleeding)	PI (% Plaque Free)	PD (mm)	CAL (mm)	Recession (mm)
Baseline								
Test	4.16±0.086	4.03±0.084	-0.13±0.044	67.66±4.665	22.32±4.300	5.74±0.073	5.27±0.092	-0.27±0.052
Control	3.93±0.083	3.72±0.079	-0.21±0.051	70.73±5.458	19.84±4.726	5.65±0.070	5.12±0.070	-0.35±0.068
Overall	4.04±0.060	3.87±0.058	-0.17±0.034	69.20±4.621	21.08±4.197	5.70±0.003	5.21±0.003	-0.31±0.002
Baseline to 3 Months								
Test	0.88±0.076	0.77±0.090	-0.06±0.047	27.88±7.054	-32.54±6.777	1.73±0.102	1.50±0.118	-0.12±0.066
Control	0.71±0.730	0.48±0.078	-0.28±0.052	35.52±6.643	-41.27±4.893	1.58±0.106	1.21±0.104	-0.32±0.075
Overall	0.80±0.053	0.63±0.060	-0.17±0.035	31.70±6.453	-36.90±3.268	1.66±0.004	1.37±0.004	-0.22±0.003
3 Months								
Test	3.27±0.071	3.25±0.077	-0.07±0.048	39.78±5.557	54.85±4.617	4.01±0.093	3.77±0.103	-0.15±0.068
Control	3.21±0.074	3.23±0.740	0.06±0.048	35.22±4.599	61.11±3.051	4.07±0.105	3.90±0.105	-0.03±0.053
Overall	3.25±0.051	3.24±0.053	0.00±0.034	37.50±4.759	57.99±3.268	4.04±0.004	3.83±0.004	-0.09±0.002
From 3 Months to 6 Months								
Test	0.25±0.56	0.31±0.074	0.05±0.049	-2.68±4.786	-4.46±5.258	0.28±0.083	0.33±0.109	0.05±0.072
Control	0.14±0.055	0.17±0.068	0.03±0.035	-2.38±5.520	-3.27±4.788	0.24±0.095	0.22±0.107	0.03±0.049
Overall	0.19±0.039	0.24±0.050	0.04±0.030	-2.53±4.940	-3.87±4.333	0.26±0.003	0.28±0.004	0.04±0.002
6 Months								
Test	3.02±0.064	2.94±0.064	-0.12±0.049	42.46±6.956	59.33±5.628	3.72±0.079	3.44±0.087	-0.20±0.061
Control	3.08±0.061	3.07±0.066	0.03±0.046	37.60±5.945	64.38±5.396	3.83±0.085	3.69±0.086	-0.06±0.058
Overall	3.05±0.044	3.05±0.046	-0.05±0.034	40.03±6.285	61.86±4.809	3.77±0.003	3.56±0.003	-0.13±0.002
From Baseline to 6 Months								
Test	1.14±0.073	1.08±0.080	-0.01±0.051	25.20±26.363	-37.00±6.509	2.02±0.099	1.83±0.107	-0.07±0.064
Control	0.85±0.070	0.65±0.075	-0.24±0.049	33.13±25.011	-44.54±6.654	1.42±0.101	1.44±0.103	-0.30±0.071
Overall	0.99±0.051	0.87±0.055	-0.13±0.035	29.17±24.178	-40.77±5.907	1.93±0.004	1.65±0.004	-0.18±0.002
